# Do we develop public health leaders?- association between public health competencies and emotional intelligence: a cross-sectional study

**DOI:** 10.1186/1472-6920-14-83

**Published:** 2014-04-17

**Authors:** Katarzyna Czabanowska, André Malho, Peter Schröder-Bäck, Daniela Popa, Genc Burazeri

**Affiliations:** 1 Department of International Health, CAPHRI, Faculty of Health, Medicine and Life Sciences, Maastricht University, P.O. Box 616, Maastricht 6200, MD, Netherlands; 2 Institute of Public Health, Faculty of Health Sciences, Jagiellonian University, Medical College, Krakow, Poland; 3 Faculty of Public Health, University of Medicine, Tirana, Albania

**Keywords:** Competencies, Emotional intelligence, Public health, Self-assessment, Survey

## Abstract

**Background:**

Professional development of public health leaders requires a form of instruction which is competency-based to help them develop the abilities to address complex and evolving demands of health care systems. Concurrently, emotional intelligence (EI) is a key to organisational success. Our aim was twofold: i) to assess the relationship between the level of self-assessed public health and EI competencies among Master of European Public Health (MEPH) students and graduates at Maastricht University, and; ii) to determine the relationship between different groups of public health competencies and specific EI skills.

**Methods:**

A cross-sectional study was conducted including all recent MEPH graduates and students from 2009–2012, out of 67 eligible candidates N = 51 were contacted and N = 33 responded (11 males and 22 females; overall response: 64.7%).Two validated tools were employed: i) public health competencies self-assessment questionnaire, and; ii) Assessing Emotions Scale.

**Results:**

Females scored higher than males in all seven domains of the self-assessed key public health competencies (NS) and emotional intelligence competences (P = 0.022). Overall, the mean value of public health competencies was the lowest in students with “staff” preferences and the highest among students with mixed job preferences (P < 0.001). There was evidence of a correlation between the overall public health competencies and the overall emotional intelligence competencies (r = 0.61, P < 0.001).

**Conclusions:**

The study shows a positive correlation between public health specific competencies and EI attributes. It can contribute to the improvement of the educational content of PH curricula by rising awareness through self-assessment and supporting the identification of further educational needs related to leadership.

## Background

The Institute of Medicine stated already years ago that the “the need for leaders is too great to leave their emergence to chance” [1] and, therefore, public health organisations should actively engage in developing leaders at every level [2]. A recent survey by the Association of Schools of Public Health in the European Region (ASPHER) asserts that European public health programmes lack modernity and do not provide adequate leadership education [3]. Professional development of public health leaders requires a form of instruction which is competency-based to help them develop the abilities to address complex and evolving demands of health care systems. Although some specific public health programmes may not include leadership training, they can still contribute to the development of leadership qualities in their students and graduates based on the public health competencies taught in their curricula. Moreover, there is a strong movement to align the curriculum as an instrument of learning to achieve requisite competencies as a key educational goal [4]. However, concerns have been raised about how to broaden leaders’ competencies, including emotional skills and intercultural communication competencies, in order to lead effectively in multinational organisations [5,6]. Emotions are important for leadership and decision-making [7]. Goleman, introducing emotional intelligence (EI), states that” without it, a person can have the best training in the world, an incisive, analytical mind, and an endless supply of smart ideas, but he or she still will not make a great leader” [8]. Moreover, leaders high in EI are the key to organisational success. Existing research also indicates that EI and intercultural consciousness have positive connotations that lead to effective cross-cultural leadership [6]. EI refers specifically to the cooperative combination of intelligence and emotion that influence one’s ability to succeed in coping with environmental demands and pressures [9].

The EI concept provides a psychometric framework for the intuitive and appealing idea that people differ in their ‘emotional skills’ and that these differences would be expected to relate to real-life outcomes such as career and relationship success [10]. When existing leaders do not possess these competencies, management can either try to develop the individuals with high potential, or implement recruitment and selection criteria that enable companies to look for and admit leaders with such attributes.

One of the methods to improve a specific subject or field is the self-assessment. This method may be viewed as “the act of evaluating one’s own level of knowledge or performance taking into account the contexts in which it occurs” [11]. Self-assessment of skills involves a high level of self-awareness and the ability to monitor one’s own learning and performance [12]. Many European university programs in public health (PH) are based on competencies which are composites of individual attributes (i.e., knowledge, skills, and attitudinal or personal aspects) that represent context-bound productivity [13] which can allow for self-assessment of students contributing to identification of gaps in knowledge and subsequent adaptation of educational programmes.

Established in 2009, the Master of European Public Health (MEPH) programme delivered by Maastricht University (the Netherlands) is a programme based on competencies. It educates students to become all-round specialists in European Public Health capable of appreciating, analysing and comprehending the impact of European and transnational integration on public health, health systems and the changing role of citizens, clients and patients. The overall aim of the programme is to provide students with state-of-the-art knowledge, academic insights and entrepreneurial skills within a broad international and European perspective. The programme allows the students to reflect on their future professional roles based on the range of values, principles and evidence that inform European Public Health practice [14]. Although leadership development has so far not been a part of the curriculum, the question arises whether we develop leaders who will be able to meet the complex challenges of contemporary public health.

In this context, our aim was to assess the relationship between the level of self-assessed public health and EI competencies among MEPH students and graduates at Maastricht University. We also aimed to determine the relationship between different groups of public health competencies, considered as important by public health employers for a successful integration on the labour market, and specific EI skills, like perceiving, using, understanding and managing emotions, and additionally assess potential age or sex differences among study participants.

## Methods

A cross-sectional study was conducted including all recent MEPH graduates from 2009/2010, 2010/2011, 2011/2012 and students of 2012/2013 (overall, 67 individuals representing 14 nationalities). Only for 51 individuals the contact details were still valid. All the eligible individuals (N = 51) were invited to participate, but only 33 of them complied (response rate was: 33/51 = 64, 7%).

### Data collection

The following validated tools were employed: i) public health competencies self-assessment questionnaire [15], and; ii) Assessing Emotions Scale [16].

The Public Health Self-assessment questionnaire was formerly used in the study measuring a self-perceived level of PH competencies among Dutch, British and Polish PH students in 2004 and to elicit the relative importance of key competencies from PH employers [15]. For this study we used two parts of this questionnaire including: questions about socio-demographic characteristics (gender and age, preferred job position) and the self-assessment of key PH competencies using two distinct questions: a) to what extent do you think you possess the key PH competences, described in the following 36 statements? using the five-point Likert scale: 1-fully disagree, 2- disagree, 3-difficult to say, 4-agree and 5- fully agree, b) To what extent does the MEPH contribute to your development of these competences? (1-very little contribution, 2-little contribution, 3-difficult to say, 4-large contribution, 5-very large contribution).

Developed by Schutte et al., “The Assessing Emotions Scale is a 33-item psychological tool measuring a self-perceived level of Emotional Quotient which attempts to assess emotional intelligence” [16]. The tool contemplates four factors: perception of emotions, managing emotions in the self, social skills or managing others’ emotions, and utilizing emotions.

The questionnaire was first reviewed by MEPH faculty, especially with respect to the list of competencies used to make sure that it reflects the current content of the programme. Two competencies were added and some were edited. Further, the questionnaire was piloted among five junior faculty members who provided comments for improvement. The tool was adapted and once more reviewed by public health experts responsible for the programme.

The questionnaire together with an explanation letter from the research coordinator was sent to all alumni and current students by e-mail. They could fill in the questions on line. Two reminders were sent to the participants within two weeks between the reminders. In case of non-response, if it was possible the individuals were approached individually, by telephone or e-mail asking for the reasons of non-responding to the survey.

### Ethical considerations

This study involved no patients or human material. The students and alumni were explained the aim and procedures of the survey and were reassured about the anonymity of the questionnaire and the subsequent aggregate data analysis. They were given the opportunity to self-select their participation. We did not obtain a written consent form from the participants. The study was conducted in compliance with the Helsinki Declaration.

### Data analysis

For the analysis of public health competencies, we used seven factors identified by Biesma et al. [15] including the following: Public health specific; Teamwork and communication; Professionalism; Advocacy, negotiation and conflict management; Project management; Deal and respond to changes; Collegiality and reflection. A summary score was calculated for each factor.

In case of the Assessing Emotions Scale, we employed Goleman’s framework which uses four characteristics:

i. Self-awareness related to identifying one’s own and other’s emotions, expressing one’s own emotions [17], and involves the capacity to recognize emotion in others’ [8].

ii. Self-management or emotional integration which is an ability to use emotions to promote emotional and intellectual growth [18], it involves the capacity of emotions to assist thinking [9].

iii. Social awareness, or emotional understanding and reasoning which reflects the capacity to analyse emotions, appreciate their probable trends over time, and understand their outcomes [9].

iv. Relationship management or emotional management defined by the ability to intentionally use emotions to influence thinking which involves an openness that allows personal and intellectual growth [19].

Mann-Whitney’s *U*-test was used to test the differences between key public health competencies and emotional intelligence competencies by sex of MEPH graduates and/or students, whereas Kruskal-Wallis test was employed to test the differences between key public health competencies by preferred job position of study participants. Conversely, Spearman’s correlation coefficients were used to assess the linear relationships between public health competencies and emotional intelligence competencies.

## Results

In this study sample, 11 (33.3%) students were males and 22 (66.7%) were females. (Table 1) There were 10 (30.3%) younger students (22–25 years) compared with 23 (69.7%) “older” students (≥26years). As for the job position, 14 (42.4%) students reported they would prefer a “staff” position, further 5 (15.2%) students would prefer a “managerial” position, 10 (30.3%) additional students would prefer a “research” position, whereas the remaining 4 (12.1%) students would prefer a mixed position (Table 1).

**Table 1:** Distribution of demographic characteristics and job preferences among study participants

**Characteristic**	**Number (N = 33)**	**Column percentage**
**Gender**		
Male	11	33.3
Female	22	66.7
**Age-group**		
22-25 years	10	30.3
≥26years	23	69.7
**Preferred job position**		
Staff	14	42.4
Management	5	15.2
Research	10	30.3
Staff and management and/or Research	4	12.1

Table 2 presents the distribution of key public health competences in male and female survey participants. The overall mean (±SD) score of public health competencies was 4.29 ± 0.29 in females vs. 4.12 ± 0.26 in males (P = 0.076). Females scored higher than males in all seven domains of the self-assessed key public health competencies, but this gender-difference was statistically significant for the “professionalism” competency only (P = 0.04). In any case, the answers of study participants refer solely to opinions about their self-perceived competencies and not their actual competencies.

**Table 2:** Distribution of key public health competencies by gender

**Public health competencies**	**Overall (N = 33)**	**Male (N = 11)**	**Female (N = 22)**	**P**^**†**^
**Public health specific**	4.17 ± 0.38^*^	4.01 ± 0.49	4.22 ± 0.31	0.466
**Teamwork and communication**	4.44 ± 0.36	4.33 ± 0.39	4.50 ± 0.34	0.264
**Professionalism**	4.41 ± 0.47	4.18 ± 0.43	4.53 ± 0.46	0.040
**Advocacy, negotiation and conflict management**	3.90 ± 0.46	3.83 ± 0.45	3.93 ± 0.48	0.715
**Project management**	4.21 ± 0.66	4.14 ± 0.67	4.25 ± 0.67	0.577
**Deal and respond to changes**	4.16 ± 0.48	4.00 ± 0.54	4.24 ± 0.44	0.301
**Collegiality and reflection**	4.29 ± 0.45	4.18 ± 0.34	4.34 ± 0.49	0.266
**Total**	4.23 ± 0.26	4.12 ± 0.26	4.29 ± 0.29	0.076

^*^Mean values ± standard deviations.

^**†**^P-values from Mann-Whitney’s *U*-test.

Table 3 presents the distribution of key public health competences by preferred job position of survey participants. Overall, the mean value of public health competencies was the lowest in students with “staff” preferences and the highest among students with mixed job preferences (P < 0.001). Furthermore, with the exception of the “colleagiality and reflection” and “deal and respond to changes”, students with mixed job preferences exhibited the highest scores, whereas students with “staff” preferences displayed the lowest scores of specific public health competencies (all p-values were statistically significant, or borderline statistically significant).

**Table 3:** Distribution of key public health competencies by preferred job position

**Public health competencies**	**Staff**	**Management**	**Research**	**Mixed**	**P**^**†**^
	**(N = 14)**	**(N = 5)**	**(N = 10)**	**(N = 4)**	
**Public health specific**	3.95 ± 0.39^*^	4.20 ± 0.17	4.36 ± 0.33	4.50 ± 0.08	0.009
**Teamwork and communication**	4.31 ± 0.33	4.33 ± 0.34	4.53 ± 0.32	4.83 ± 0.33	0.088
**Professionalism**	4.14 ± 0.43	4.20 ± 0.45	4.70 ± 0.29	4.92 ± 0.17	0.002
**Advocacy, negotiation and conflict management**	3.75 ± 0.36	4.07 ± 0.48	3.82 ± 0.51	4.46 ± 0.31	0.061
**Project management**	3.86 ± 0.69	4.40 ± 0.55	4.40 ± 0.52	4.75 ± 0.50	0.049
**Deal and respond to changes**	3.88 ± 0.45	4.20 ± 0.29	4.53 ± 0.39	4.17 ± 0.43	0.015
**Collegiality and reflection**	4.07 ± 0.43	4.00 ± 0.35	4.65 ± 0.34	4.50 ± 0.07	0.003
**Total**	3.99 ± 0.19	4.20 ± 0.26	4.42 ± 0.14	4.59 ± 0.08	<0.001

^*^Mean values ± standard deviations.

^**†**^P-values from Kruskal-Wallis test.

Table 4 presents the distribution of emotional intelligence competences by gender of survey participants. Overall, the mean value of emotional intelligence competences was higher in female students than in male students (4.01 vs. 3.67, P = 0.022). Also, the mean value of each specific emotional intelligence competence was higher in females than in males, but these findings were borderline statistically significant for “self-awareness” and “social awareness” domains only.

**Table 4:** Distribution of emotional intelligence competencies by gender

**Emotional intelligence competencies**	**Total (N = 33)**	**Males (N = 11)**	**Females (N = 22)**	**P**^**†**^
**Self-awareness**	3.75 ± 0.61^*^	3.39 ± 0.73	3.93 ± 0.46	0.066
**Self-management**	3.93 ± 0.42	3.78 ± 0.38	4.00 ± 0.43	0.161
**Social-awareness**	3.91 ± 0.46	3.72 ± 0.38	4.01 ± 0.48	0.071
**Relationship management**	4.01 ± 0.48	3.82 ± 0.49	4.09 ± 0.45	0.114
**Total**	3.89 ± 0.69	3.67 ± 0.35	4.01 ± 0.34	0.022

^*^Mean values ± standard deviations.

^**†**^P-values from Mann-Whitney’s U-test.

Table 5 presents a correlation matrix of key public health competencies and emotional intelligence competencies. There was evidence of a significant quite strong correlation between the overall public health competencies and the overall emotional intelligence competencies (Spearman’s rho = 0.61, P < 0.001). The overall score of emotional intelligence competencies was quite highly correlated with the “public health specific” domain (r = 0.66, P < 0.001), and less so with the “project management” competency (P = 0.28, P = 0.121). Conversely, the overall score of public health competencies was mostly correlated with the “self-awareness” domain (r = 0.53, P = 0.002) and less so with the “self-management” competency (r = 0.29, P = 0.097).

**Table 5:** Correlation matrix between public health competencies and emotional intelligence competencies

	**Self-awareness**	**Self-management**	**Social-awareness**	**Relationship management**	**Total**
**Public health specific**	0.61 (<0.001)^*^	0.39 (0.026)	0.56 (0.001)	0.36 (0.042)	0.66 (<0.001)
**Teamwork and communication**	0.31 (0.080)	0.43 (0.012)	0.27 (0.132)	0.18 (0.326)	0.34 (0.055)
**Professionalism**	0.34 (0.053)	0.04 (0.840)	0.26 (0.142)	0.18 (0.311)	0.33 (0.059)
**Advocacy, negotiation and conflict management**	0.27 (0.125)	0.21 (0.246)	0.31 (0.079)	0.24 (0.183)	0.34 (0.050)
**Project management**	0.13 (0.482)	0.06 (0.732)	0.30 (0.095)	0.18 (0.327)	0.28 (0.121)
**Deal and respond to changes**	0.28 (0.117)	0.05 (0.766)	0.25 (0.158)	0.29 (0.108)	0.39 (0.023)
**Collegiality and reflection**	0.44 (0.010)	0.29 (0.100)	0.39 (0.023)	0.12 (0.501)	0.48 (0.005)
**Total**	0.53 (0.002)	0.29 (0.097)	0.51 (0.003)	0.34 (0.050)	0.61 (<0.001)

^*^Spearman’s correlation coefficients and their respective p-values (in parentheses).

## Discussion

The study shows a positive correlation between public health specific competencies and EI attributes which are the foundations for personal and work success [8]. More specifically public health competencies correlate with self -awareness and less with project management and self-management which are more technical, employment related skills. According to the model of EI which serves as the basis for University of North Carolina (UNC) Leadership Development Institutes [20] p.243” self-awareness is a component of personal competence, constituting the foundation of EI which is characterised by knowing, understanding and expressing oneself” [20] p. 250. Personal competence creates leverage points for the development of other, more sophisticated skills such as social competence including social awareness and relationship management which is a crucial leadership skill [20-22]. One might say that the specificity of PH training which among others focuses on such competencies as: ability to access and understand: information regarding environmental and biochemical influences on health, epidemiological research information, socio-economic, behavioural factors that impact health, ability to gather and utilise research information to analyse and diagnose PH problems [15] can contribute to the development of self-awareness among the MEPH students and graduates. It can be also reinforced by the use of problem-based and self-directed teaching and learning approaches which strongly rely on collaboration of peers and their feedback [23]. The strong positive correlation between PH specific competencies and EI may imply that the MEPH studies provide a good foundation for further development of more sophisticated leadership skills related to cultural awareness, empathy and relationship management in the form of additional leadership modules, continuing professional development or work-related training.

Although female participants scored higher than men in all public health competencies it was professionalism which was statistically significant. Epstein argues that professional competence is more than demonstration of isolated competencies [24] he further states “that professional competence is more than factual knowledge and ability to solve problems with clear-cut solutions: it is defined by the ability to manage ambiguous problems, tolerate uncertainty and make decisions with limited information” [24]. Such an argumentation is in line with the current influential WHO document which states that particular type of leadership required is not of a traditional command and control variety but rather akin to what has been termed “adaptive” leadership: leading in contexts where there is considerable uncertainty and ambiguity, and where there is often imperfect evidence and an absence of agreement about both the precise nature of the problem and the solutions to it [25].

While Biesma and colleagues [15] showed that PH employers consider as highly important the possession of the key competency “teamwork and communication” by PH graduates, our study participants scored highest in all factors of public health specific competencies. Similar results were obtained in other studies measuring skills and educational mismatch in health graduates [26-28].

The study participants seemed to favour staff and research positions for the future public health jobs. Interestingly, those who preferred staff positions scored lower on public health specific competencies and those who favoured mixed positions scored higher apart from collegiality and reflection and deal and respond to changes. This finding may point to the fact that participants who preferred a mixed position have internalized a more comprehensive/holistic perspective (alias, set of core competencies) as compared to the more pragmatic individuals who seek for a specific staff position in their future work.

### Limitations

Although our study seems to imply that EPH program at Maastricht University contributes to the development of EI especially personal competence among the graduates, there are limitations to this study. Firstly, the sample size was limited to the students attending this particular programme which is unique in Europe and slightly different results may be obtained if graduates of other public health programmes were included in the study for a comparison. Moreover, “recent reviews of self-assessment in health professions raise questions about ability of professionals to generate accurate judgements of their own performance” [29]. This may be even of more concern when the area of assessment is related to value judgements such as professionalism [29]. In addition, our cross-sectional findings do not distinguish whether there is a real sex difference in competences’ level, or in self-assessment (i.e. perceived level of competencies). Thus, female participants may have, on average, higher competences’ score than their male counterparts, or they may simply self-assess their competences as higher.

Another potential limitation may be the fact that graduates of four generations were mixed together in the current analysis due to the small sample size. Future and larger studies on this topic should assess whether younger students differ in their competences’ level from graduates of previous generations who are expected to be already employed and have gained experience during 1–3 years after graduation. In particular, it might be important to assess competences’ level regarding the preferred job positions between younger students and graduates of previous generations.

Finally, surveys are subject to social desirability and bias [30].

There may also be educational benefits from this study. On one hand it stirs the reflection on the content and process of learning in the PH course both among participants who can monitor their own learning [31] and teachers and on the other it sets direction related to the design of the PH leadership training and its inclusion in the existent curriculum. It may also lead educators to give more attention to stimulating students’ awareness and understanding of their peers and the future need of understanding others as a premise for effective leadership. The question remains whether the teaching methods used in MEPH make reference to emotional intelligence and stimulate the development of PH leaders, or at least push them to think in a different way, as leadership is intrinsically an emotional process.

## Conclusion

In spite of its limitations, our study makes an important contribution to the understanding of the process of developing leaders through PH education by showing positive relation between PH and EI competencies. It can contribute to the improvement of the educational content of PH curricula by rising awareness through self-assessment and supporting the identification of further educational needs related to leadership.

## Abbreviations

ASPHER: Association of schools of public health in the European Region; EI: Emotional intelligence; MEPH: Master of European public health; PH: Public health.

## Competing interests

The authors declare that they have no competing interests.

## Authors’ contributions

KC developed the study design, contributed to the analysis, drafting of the manuscript and prepared it for submission, AM contributed to the study design, collected data and contributed to the analysis, PSB critically reviewed the manuscript and supported data collection, DP critically reviewed the article and supported its development, GB supported the data analysis and development of the results section as well as critically reviewed the article. All authors have read and approved the final manuscript.

## Pre-publication history

The pre-publication history for this paper can be accessed here:

http://www.biomedcentral.com/1472-6920/14/83/prepub
